# Detection of Group 1 Coronaviruses in Bats in North America

**DOI:** 10.3201/eid1309.070491

**Published:** 2007-09

**Authors:** Samuel R. Dominguez, Thomas J. O’Shea, Lauren M. Oko, Kathryn V. Holmes

**Affiliations:** * University of Colorado Health Sciences Center, Aurora, Colorado, USA; † US Geological Survey, Fort Collins, Colorado, USA; 1 These authors contributed equally to this article.

**Keywords:** Coronavirus, bat coronavirus, coronavirus group 1, bats, phylogeny of bat coronaviruses, wildlife viral surveillance, research

## Abstract

The epidemic of severe acute respiratory syndrome (SARS) was caused by a newly emerged coronavirus (SARS-CoV). Bats of several species in southern People’s Republic of China harbor SARS-like CoVs and may be reservoir hosts for them. To determine whether bats in North America also harbor coronaviruses, we used reverse transcription–PCR to detect coronavirus RNA in bats. We found coronavirus RNA in 6 of 28 fecal specimens from bats of 2 of 7 species tested. The prevalence of viral RNA shedding was high: 17% in *Eptesicus fuscus* and 50% in *Myotis occultus*. Sequence analysis of a 440-bp amplicon in gene *1b* showed that these Rocky Mountain bat coronaviruses formed 3 clusters in phylogenetic group 1 that were distinct from group 1 coronaviruses of Asian bats. Because of the potential for bat coronaviruses to cause disease in humans and animals, further surveillance and characterization of bat coronaviruses in North America are needed.

## Abstract 2

Bats of 2 species harbor group 1 coronaviruses.

Emerging diseases are frequently zoonoses caused by RNA viruses (*1*,*2*). Defense against emerging infectious diseases, identification of reservoirs for such viruses, surveillance for host-jumping events, and elucidation of viral and host factors that may facilitate such events are warranted. The epidemic of severe acute respiratory syndrome (SARS) in 2002–2003 was caused by a newly emerged zoonotic coronavirus (SARS-CoV) (order Nidovirales, family *Coronaviridae*, genus *Coronavirus*). Other coronaviruses have also jumped to new host species and caused emerging diseases. For example, porcine epidemic diarrhea virus emerged in European pigs from an unknown host species during the late 1970s and caused severe enteric disease (*3*). Human coronavirus OC43 is believed to have been derived from bovine coronavirus (*4*). In addition, the genomes of canine and feline coronaviruses can recombine in vivo and have developed into different biotypes that are serially transmissible in their new host species (*5*).

SARS-CoV entered the human population as a result of a zoonotic transmission in southern People’s Republic of China in 2002. Epidemiologic studies demonstrated that the first human cases of SARS were caused by coronaviruses closely related to viruses found in masked palm civets (*Paguma larvata*) and raccoon dogs (*Nyctereutes procyonoides*) in live animal markets (*6*). Subsequently, surveys of coronaviruses in domestic animals, livestock, poultry, and wildlife were conducted in Southeast Asia to identify the reservoir(s) of SARS-CoV. On the basis of low prevalence of SARS-like CoVs in wild and farmed masked palm civets, these animals are now believed to be an intermediate host rather than a primary reservoir for SARS-CoV (*7*). During these surveys, a wide variety of coronaviruses were detected in many bat species in Asia (*8*–*11*).

Horseshoe-nosed bats of several species (suborder Microchiroptera, family Rhinolophidae, genus *Rhinolophus*) from different locations in southern People’s Republic of China and the Hong Kong Special Administrative Region were found to be infected with SARS-like CoVs, and some of the bats had antibodies to these newly recognized coronaviruses (*10*,*12*). Phylogenetic analysis of the complete genome sequences of the bat SARS-like CoVs showed that they form a large and diverse clade within phylogenetic group 2b (also called group 4), which includes SARS-CoVs from palm civets and humans obtained during the 2002–2003 outbreak (*10*,*12*,*13*). Thus, the virus responsible for the SARS pandemic may have originated from bats, perhaps with the palm civet as an intermediate host. In addition to SARS-like CoVs, RNAs of many other viruses belonging to coronavirus groups 1 and 2a, and proposed new group 5, were detected in several species of Asian bats (*8*,*9*,*14*,*15*). To date, no infectious bat coronavirus has been isolated in cell culture.

We investigated whether bats in North America also harbor coronaviruses. To our knowledge, we provide the first evidence of coronaviruses in bats in the Western Hemisphere. We studied oral, anal, and fecal specimens from 57 bats in the Rocky Mountain region and detected coronavirus RNA in 6 of 28 fecal specimens from 2 of 7 bat species tested. Limited sequence analysis showed that these viruses are in phylogenetic group 1 and that they differ from group 1 coronaviruses of Asian bats.

## Materials and Methods

### Sample Collection

Bats were sampled at 4 sites in the Rocky Mountain region in August 2006. At sites 1 and 2, bats of 2 species were sampled in colonies inhabiting 2 buildings 480 km apart on opposite sides of the continental divide of the Rocky Mountains. Eight occult myotis (*Myotis occultus*) and 1 Brazilian free-tailed bat (*Tadarida brasiliensis*) were captured in mist nets as they emerged from a roost in a building in Mancos in Montezuma County in southwestern Colorado (site 1) at dusk on August 19. This species was previously thought to be conspecific with the little brown bat (*M*. *lucifugus*) that is common throughout North America (*16*). Big brown bats (*Eptesicus fuscus*) were sampled at a roost in a building in Fort Collins in Larimer County in north-central Colorado (site 2) on August 7. Other bats (n = 27) were sampled at sites 3 and 4 incidental to ongoing, unrelated bat faunal surveys. One western small-footed myotis (*M*. *ciliolabrum*) and 1 long-eared myotis (*M*. *evotis*) were captured in mist nets over water on August 8 at Soapstone Prairie Natural Area in Larimer County (site 3). Four big brown bats, 3 long-eared myotis, 8 occult myotis, 1 Brazilian free-tailed bat, 7 long-legged myotis (*M*. *volans*), and 2 silver-haired bats (*Lasionycteris noctivagans*) were trapped in mist nets during the nights of August 14–20 as they drank or foraged near open water at 2 sewage treatment lagoons (9 km apart) (site 4) in Montezuma County, Colorado. Bats were captured under authority of a Colorado Division of Wildlife Scientific Collection License following procedures approved by the Institutional Animal Care and Use Committee of the US Geological Survey, Fort Collins Science Center. Typically, each bat was sampled within 5–10 minutes of capture and then released.

Whenever possible, 3 sample types were taken from each bat (Table). Sterile calcium alginate swabs were used for oral or anal area samples that were immediately placed into 2 mL of RNAlater (Ambion, Austin, TX, USA). Fecal samples were collected if the bat produced a fresh bolus during handling. Disposable latex gloves were changed between samples, and multiple forceps used to collect fecal boluses were rinsed, wiped in ethanol, and air-dried between samples. Samples were numbered, kept in a cooler in the field, stored at 4°C, and delivered to the laboratory on August 28.

**Table:** Reverse transcription–PCR analysis of coronaviruses in Rocky Mountain bats*

Location	Bat species	No. bats tested	Anal (positive/total)	Oral (positive/total)	Fecal (positive/total)	Positive samples
Site 1	*Tadarida brasiliensis*	1	ND	0/1	ND	
	*Myotis occultus*	8	0/3	0/6	1/2	Bat 27
Site 2	*Eptesicus fuscus*	21	0/21	ND	1/3	Bat 65
Site 3	*M*. *ciliolabrum*	1	ND	ND	0/1	
	*M*. *evotis*	1	ND	ND	0/1	
Site 4	*E*. *fuscus*	4	0/1	0/2	0/3	
	*M*. *evotis*	3	0/2	0/1	0/1	
	*Lasionycteris noctivagans*	2	ND	0/2	0/2	
	*M*. *volans*	7	0/2	0/4	0/6	
	*T*. *brasiliensis*	1	ND	0/1	0/1	
	*M*. *occultus*	8	ND	0/5	4/8	Bats 3, 6, 11, 48
	Total	57	0/29	0/22	6/28	

*ND, not determined (no samples available for analysis).

### RNA Extraction and Reverse Transcription (RT)

RNA from 140 μL of each of the 79 samples was extracted by using the QIamp viral RNA mini kit (QIAGEN, Valencia, CA, USA) following the manufacturer’s instructions. Extracted RNA was eluted in 50 μL of RNase-free water and stored at –80°C. We used Moloney murine leukemia virus reverse transcriptase (Invitrogen, Carlsbad, CA, USA) with random hexamers in a 20-μL reaction to generate cDNAs by using 10 μL of RNA as a template according to the manufacturer’s instructions. All samples were extracted and analyzed in triplicate. RT products were stored at –20°C.

### PCR and Sequencing

All samples were screened by PCR and nested PCR. On the basis of previous reports, PCR with a pair of consensus primers that target a highly conserved region of coronavirus gene *1b* was used to screen the cDNA samples (*8*). Three microliters of cDNA was amplified in a 50-μL reaction containing 1.5 mmol/L MgCl_2_, 0.2 mmol/L deoxynucleoside triphosophates, 2.5 U of HotStarTaq (QIAGEN), and 0.2 μmol/L of primers 1 and 2: 5′-GGTTGGGACTATCCTAAGTGTGA-3′ (primer 1) and 5′-CCATCATCAGATAGAATCATCATA-3′ (primer 2) by using the following PCR program: 15 min at 95°C; 45 cycles for 1 min at 95°C, 1 min at 48°C, and 1 min at 72°C; and 10 min at 72°C.

For nested PCR, 5 μL from each PCR was amplified in a 50-μL reaction with primer 2 and primer 3 (5′-GTTGTACTGCTAGTGACAGG-3′), an internal primer based on nucleotide sequences of the PCR amplicons by using 40 cycles of the same PCR program. All RT-PCRs were conducted in an enclosed nucleic acid workstation equipped with a UV light (Clone Zone; USA Scientific, Ocala, FL, USA) in a room separate from the main laboratory. Water controls in all RT-PCRs did not show false-positive results. To overcome possible PCR inhibitors in fecal samples, PCR was performed both on the cDNA and on a 1:10 dilution of the cDNA. Amplicons were analyzed by agarose gel electrophoresis. For each positive specimen, amplicons from 2 independent RT-PCRs were sequenced on an ABI 3730 DNA sequencer (Applied Biosystems, Foster City, CA, USA) at the University of Colorado Health Science Center Cancer Center DNA Sequencing and Analysis Core. Numbered specimens were then correlated with lists of bat samples.

### Data Analysis

Viral sequences were analyzed and aligned by using ClustalW (http://workbench.sdsc.edu). Phylogenetic trees were constructed by using the neighbor-joining method in the program PAUP* version 4.0 (Sinauer Associates, Inc., Sunderland, MA, USA) rooted with porcine respiratory and reproductive syndrome virus (GenBank accession no. NC_001961). Sequences used for alignment were AF304460 (HCoV-229E), AY567487 (HCoV-NL63), DQ648858 (BtCoV 512), AY594268 (BtCoV HKU2), DQ249224 (BtCoV HKU6), DQ249226 (BtCoV HKU7), and DQ249228 (BtCoV HKU8). The deduced sequences from this study were deposited in GenBank under accession nos. EF544563–EF544568.

## Results

### Identification of Rocky Mountain Bat Coronaviruses (RM-Bt-CoVs)

A total of 79 samples (28 fecal samples, 29 anal swab specimens, and 22 oral swab specimens) were collected from 57 bats of 7 species in 4 locations in the Rocky Mountain region during a 2-week period in August 2006 (Table). PCR amplicons that target a conserved region in gene *1b* containing the RNA-dependent RNA polymerase common to all coronaviruses were detected in reversed-transcribed RNA from 6 of the 79 samples. All samples positive for coronavirus RNA were from the 28 fecal samples tested (Table). None of the anal region or oral swab specimens were positive for coronavirus RNA.

Despite the small number of bats sampled, there was a high prevalence of coronavirus RNA shedding in fecal samples of 2 species of bats. Five (50%) of 10 fecal samples from occult myotis and 1 (17%) of 6 fecal samples from big brown bats were positive for coronavirus in screening tests. The 1 coronavirus-positive sample from big brown bat (bat sample 65) was from feces of 1 (33%) of 3 big brown bats sampled at site 2 in north-central Colorado, whereas the positive samples from the occult myotis (bat samples 3, 6, 11, 27, and 48) were from sites 1 and 4 in southwestern Colorado, ≈480 km from site 2 (Table). Most of the fecal samples were only positive in the PCRs with cDNA diluted 1:10, which suggested that PCR inhibitors were present in feces. In addition, most of the samples were positive only in the nested PCRs, which indicated that either the RNA was present in small amounts or that the primers used were not an optimal match for these viruses.

### Phylogenetic Analysis of RM-Bt-CoVs

**Figure 1:**
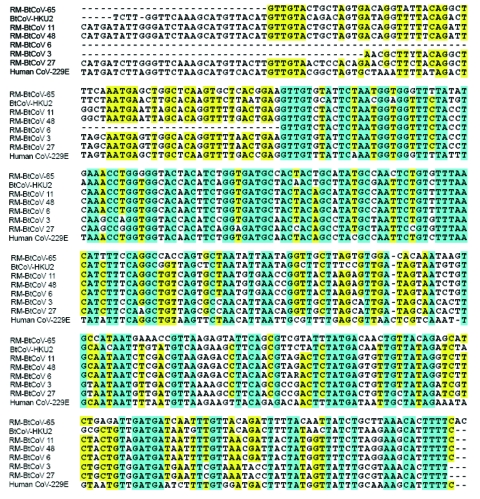
Nucleotide sequence alignment of amplicons from a 440-nt region of gene *1b* of Rocky Mountain bat coronaviruses (RM-Bt-CoVs) compared with group 1 coronaviruses of Asian bats (BtCoVs) and human coronavirus 229E. Identical residues are shaded in blue and similar residues are shaded in yellow. Hyphens indicate positions where sequences are not available.

**Figure 2:**
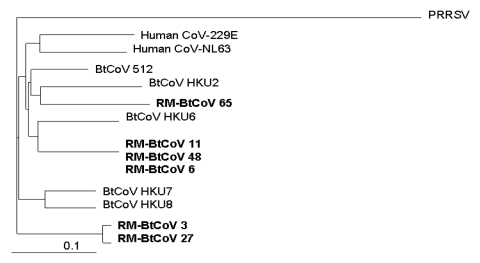
Phylogenetic relationships based on a 440-nt sequence in a conserved region of gene *1b* of Rocky Mountain bat coronaviruses (RM-Bt-CoVs) (shown in **boldface**), group 1 coronaviruses of Asian bats (BtCoVs), and human coronaviruses 229E and NL63. Porcine respiratory and reproductive syndrome virus (PRRSV) was used as the outgroup to root the tree. Scale bar at the lower left indicates 0.1 nucleotide substitutions per site.

A 440-nt sequence in the RNA-dependent RNA polymerase region of gene *1b* was amplified by RT-PCR from the 6 positive samples. Analysis of nucleotide sequences of these amplicons showed that all 6 RM-Bt-CoVs are members of coronavirus group 1 (Figure 1). Although these sequences were similar to those published for Asian bat group 1 coronaviruses, there was enough dissimilarity in this highly conserved region to suggest that the Rocky Mountain specimens represent unique coronaviruses (*8*,*9*). Phylogenetic analysis of this region of gene 1b suggests that the RM-Bt-CoVs cluster in 3 subgroups within group 1. Three of the 5 specimens from the occult myotis (samples 6, 11, and 48) were in 1 cluster and the other 2 (samples 3 and 27) formed a second cluster within group 1 coronaviruses. The 1 specimen from the big brown bat (sample 65) was a more distantly related group 1 coronavirus (Figure 2).

## Discussion

To our knowledge, this is the first report of coronaviruses in bats in the Western Hemisphere. With >1,100 species, bats are among the most divergent and widely distributed nonhuman mammals (*17*). Bats are reservoirs for rabies virus and other lyssaviruses and were recently shown to be reservoirs for other important emerging viruses. Old World fruit bats (family Pteropodidae) are reservoirs for Hendra virus, which caused small outbreaks of severe respiratory illnesses in horses and humans in Australia (*18*–*24*) and Nipah virus, which caused large outbreaks of lethal encephalitis and respiratory illnesses in humans and pigs in Malaysia and Singapore (*25*–*28*). Old World fruit bats may also be the long-sought reservoir hosts for Ebola and Marburg viruses (*29*,*30*). More than 60 different RNA viruses have been isolated from and detected in bats, which play important roles in maintaining and transmitting zoonotic viruses (*31*–*33*).

The need for understanding the ecology and evolution of coronaviruses in wildlife was highlighted by the observation that SARS-CoVs that caused 4 sporadic human cases of SARS in 2003–2004 were more closely related to viruses from palm civets found in 2004 than to the human epidemic strain of SARS-CoV (*34*). The gene encoding the viral spike glycoprotein that binds the virus receptor human angiotensin-converting enzyme 2 was one of the fastest-adapting genes of SARS-CoV during the 2002–2003 epidemic. Nonsynonymous amino acid substitutions in the spike protein that were selected during the epidemic optimized binding of the spike to its human receptor and enhanced human-to-human transmission (*34*,*35*). Sequencing of SARS-CoV genomes during and after the epidemic suggests that multiple independent species-jumping events of SARS-CoV from animals to humans have occurred.

Although all samples we tested were from apparently healthy wild bats, a high prevalence of coronavirus RNA was detected in 2 of the 7 species of bats tested. Five (50%) of 10 occult myotis and 1 (17%) of 6 big brown bats tested contained low levels of coronavirus RNA in feces. No coronavirus RNA was detected in the oral or anal region swabs tested. Similarly in Asian bats, coronavirus RNA was found in a higher percentage of fecal samples than saliva samples (*8*,*9*,*14*). Thus, bats may be persistently infected carriers that shed low levels of coronaviruses in feces. Persistent fecal shedding of coronaviruses has also been detected in pigs, cats, dogs, and cattle (*36*). The mechanisms for persistent fecal shedding of viruses in bats without apparent disease have not yet been determined (*32*,*33*).

No bat of any species occurs in both the Eastern and Western Hemispheres (*37*). Therefore, it is of great interest that group 1 coronaviruses have now been found in bats in North America as well as in Asia. Comparison of the nucleotide sequences of related coronaviruses from different species of bats on different continents is likely to provide information about coronavirus evolution. Figure 2 shows the phylogeny of RM-Bt-CoVs in relation to group 1 coronaviruses from Asia on the basis of the 440-nt amplicon in gene *1b*. Bats of the genera *Myotis* and *Eptesicus* are in the family Vespertilionidae, which has diversified into many different species in the Eastern and Western Hemispheres (*17*). Amplicons of 3 of the 5 coronaviruses (samples 6, 11, and 48) from occult myotis in Colorado have the highest nucleotide sequence identity with the HKU6 bat coronavirus found in an Asian bat of the same genus but a different species, Rickett’s big-footed myotis (*M*. *ricketti,* subfamily Myotinae) (*11*,*17*). The coronavirus RNA in the big brown bat (sample 65) from Colorado (subfamily Vespertilioninae) was most similar to HKU2 bat coronavirus found in Asian bats in the family Rhinolophidae (*11*) (Figure 2). Rhinolophid bats are not found in the Western Hemisphere and are phylogenetically far removed from the big brown bat (*37*,*38*).

In our small, initial study of coronaviruses in North American bats, samples were restricted in size, location, and variety of bat species, and we found only group 1 coronaviruses. When larger numbers of bats and additional bat species in North America are studied, additional bat coronaviruses with complex phylogenetic attributes, biogeographic patterns, and perhaps epizootiologic attributes may be discovered. For example, determining if North American bat coronaviruses are species-specific will provide useful information. In Asia, different species of bats roosting in the same cave host different coronaviruses (*9*). However, bats of 1 species can also harbor different types of coronaviruses at different geographic locations (*9*).

A recent analysis of genome sequences of coronaviruses of bats, other animals, humans, and birds suggested that bats may be the original hosts from which all coronavirus lineages were derived (*15*). We find this hypothesis intriguing, in light of the high prevalence and diversity of coronaviruses in bats in North America found in our initial small survey. The North American species of bats found to harbor group 1 coronaviruses commonly roost in buildings inhabited by humans (*39*), which provides ecologic overlap between these bats and humans. Before the SARS epidemic of 2002–2003, only 2 coronaviruses, HCoV-229E and HCoV-OC43, were known to cause human disease, primarily mild upper respiratory tract infections. In contrast, SARS-CoV caused severe lower respiratory tract disease with a death rate of 10%. Recently, 2 additional human coronaviruses, HCoV-NL63 and HCoV-HKU1, were discovered and found to cause both upper and lower respiratory tract infections worldwide (*40*).

It is possible that another epidemic caused by an emerging coronavirus could occur in the future. As in the SARS epidemic, bats could play a role in future emergence of coronaviruses in humans or other species. Isolation of infectious bat coronaviruses and elucidation of their host ranges, receptor specificities, and genetic diversity will greatly aid in our understanding of their potential for emergence.
